# Fairness in the Use of Information About Carriers of Resistant Infections

**DOI:** 10.1007/978-3-030-27874-8_15

**Published:** 2020-04-06

**Authors:** John G. Francis, Leslie P. Francis

**Affiliations:** 2 Monash Bioethics Centre, Monash University, Melbourne, VIC Australia; 3 Monash Bioethics Centre, Monash University, Melbourne, VIC Australia; 4 Department of Political Science, University of Utah, Salt Lake City, USA; 5 College of Law & Department of Philosophy, University of Utah, Salt Lake City, USA

**Keywords:** Fairness, Data use, Privacy, Surveillance

## Abstract

One standard menu of approaches to the prevalence of anti-microbial resistance diseases is to enhance surveillance, fund research to develop new antimicrobials, and educate providers and patients to reduce unnecessary antimicrobial use. The primarily utilitarian reasoning behind this menu is unstable, however, if it fails to take fairness into account. This chapter develops an account of the fair uses of information gained in public health surveillance. We begin by sketching information needs and gaps in surveillance. We then demonstrate how analysis of information uses is incomplete if viewed from the perspectives of likely vectors of disease who may be subjects of fear and stigma and likely victims who may be coerced into isolation or quarantine. Next, we consider aspects of fairness in the use of information in non-ideal circumstances: inclusive participation in decisions about information use, resource plans for those needing services, and assurances of reciprocal support. Fairness in information use recognizes the ineluctable twinning of victims and vectors in the face of serious pandemic disease.

As many chapters in this volume emphasize, the prevalence of anti-microbial resistant diseases and the comparative paucity of available treatments presents a public health crisis. One standard menu of approaches to this crisis is to enhance surveillance to gain information needed to identify potential disease vectors and to ascertain likely modes of transmission; to fund research to develop new treatments and antimicrobials; and to intervene through education, treatment, and careful stewardship of the existing antimicrobials that retain some efficacy. This combination of approaches is founded primarily in utilitarian reasoning, attempting to achieve the best possible mitigation of the current crisis in the hopes that effective new treatment methods may soon become available.

Such utilitarian reasoning is not entirely stable in practice, however. On the one hand, when the prospects of exposure to untreatable and potentially fatal disease appear imminent, fear may become the overriding reaction to those who are identified as ill. The result may be forms of coercion against people suspected of being vectors of disease that appear prudential in the short term but that are insufficiently grounded in science and potentially counter-productive in the longer term. People may hide to avoid disclosure and deleterious consequences of over-regulation may lead to under-regulation. Recent examples include demands to compel isolation of people believed to have been exposed to Ebola or for banning travel from regions where outbreaks of conditions such as Ebola or Zika have been identified. On the other hand, concerns for victims may generate outpourings of resources for treatment, calls for investment in public health resources in underserved areas, and renewed emphasis on privacy protections. These too may be counterproductive if they result in confusion and waste of resources or multiple conflicting strategies. The upshot may be policies that oscillate between treating people as vectors and treating them as victims but without significant or coordinated progress against the problem of resistance.

Each of these perspectives—victim-hood and vector-hood—is morally important. But in our judgment analysis that is limited to these perspectives is incomplete in its failure to take certain considerations of fairness into account. Our specific focus here is the use of information, but similar points could be made about other types of resources as well. Collection, uses, and access to information, we contend in what follows, must be rooted in the effort to make progress against serious public health problems in a manner that is reasonably fair under the circumstances. This requires not only concern for people as victims and vectors but concerns about how the impact of policies are distributed and foster cooperative connections in both the shorter and the longer term.

## The Important Roles of Information

Traditional public health surveillance methods are both individual and population based. Where particular individuals are concerned, the role of information is primarily to enable strategies to interrupt disease transmission. Case identification, case reporting, contact tracing, treatment if possible, and education and intervention if needed to prevent transmission come to the fore. At every stage, information is critical. If individuals with transmissible disease are unknown or cannot be located, efforts to interrupt transmission will fail. Efforts will also fail if information is not transmitted to those who are capable of acting, whether they be authorities designated to enforce quarantine or isolation or health care personnel equipped to offer treatment or prophylaxis. Education requires information, too, about where to direct educational efforts and what these efforts might contain. Importantly, if people who might suffer exposures are insufficiently informed about the likelihood and seriousness of contagion and the need for precautions, they may unwittingly become infected vectors as well as victims themselves. Such was the case for health care workers during the SARS epidemic of 2003 and for many during the Ebola epidemic of 2014.

Information gleaned in population-level surveillance plays many additional important roles in addressing the problem of anti-microbial resistance. A longstanding recommendation of the WHO, codified in the World Health Regulations that entered into force in 2007 in article 44, is international cooperation in the development of surveillance capacities for the identification of potential global health emergencies of international concern (WHO 2005). Surveillance can help to identify rates of incidence and prevalence of resistant disease. Testing samples can yield information about histories and patterns of disease spread. Samples also can be used to identify biological characteristics of resistant infectious agents that may be helpful in developing methods of treatment or identifying new anti-microbial agents.

Population level surveillance can be targeted to identifying the incidence and prevalence of resistant disease in particular geographical areas. Gonorrhea is an example. There were 78.3 million estimated new cases of gonorrhea worldwide in 2012; the highest number occurred in low-income areas of the western Pacific. Resistant disease has become increasingly prevalent, especially in these areas and among groups such as sex workers and truck drivers (Unemo et al. 2017). Extensively drug-resistant (XDR) gonorrhea cases also have appeared in Spain and in France, although these strains do not appear to have spread, possibly because they are less hardy and so less likely to be passed on. However, significant resistance may not be detected because of “suboptimal antimicrobial resistance surveillance in many settings” (Unemo et al. 2017). A recent international panel reviewing resistant gonorrhea recommends strategies of case management, partner notification, screening (especially of sex workers and men having sex with men), and evidence-based treatment (Unemo et al. 2017); these recommendations are based on surveillance data.

Population-level surveillance information may also be useful in identifying risks associated with providing humanitarian treatment. Over 30,000 young people wounded in the Libyan civil war that began in 2011 were evacuated elsewhere for treatment. Concerns arose that many of these patients were recognized to carry with them resistant organisms—thus bringing along with their needs for treatment risks to other patients being treated in the host facilities (Zorgani and Ziglam 2013). Institutions accepting these patients were informed of this risk so that they could take appropriate precautions. Libya itself was identified as a region with high prevalence of resistant organisms, despite the limited surveillance capacities in that conflict-torn nation. Recommendations included improving surveillance in Libya—which lacks a national surveillance system—and implementation of infection prevention measures in Libyan hospitals.

 Surveillance is also used to identify practices that might contribute to the development of resistance. Use of antimicrobials in agriculture is one area of inquiry, although its precise contribution to the problem is not easy to quantify (e.g. Hoelzer et al. 2017). There have been many studies of problematic prescribing practices among physicians in the US (Wigton et al. 2008), Europe (e.g. Jørgensen et al. 2013), Asia (Lam and Lam 2003), and elsewhere (Trap and Hansen 2002), along with efforts to educate physicians about appropriate antimicrobial use.

Ever since the recognition grew that crowds celebrating the return of soldiers from World War I had created a ready opportunity for transmission of the Spanish influenza, epidemiologists have observed the potential health risks of large gatherings that concentrate people together, even for brief periods of time. Examples include music festivals, major sporting competitions, other large festivals, and religious gatherings such as the Hajj or other pilgrimages. The largest estimated gathering is the periodic Kumbh Mela pilgrimage in which Hindus come together to bathe in a sacred river such as the Ganges; over 40 million people, drawn largely from the Indian subcontinent but increasingly international, attend the event (Gautret and Steffen 2016). The largest annual gathering of pilgrims is the Hajj at Mecca which draws over two million people; the Fifth Pillar of Islam is the obligation to undertake the once in a lifetime journey for those who can physically or financially afford to do so. With such great numbers of people together for sustained periods of time, there is a risk of disease outbreaks and the spread of resistant infections. Such events may strain existing sanitation systems or health care facilities if people become ill. Crowding and inadequate facilities contribute to the potential for disease outbreaks (Gautret and Steffen 2016). These events draw people from around the globe and thus may result in the international spread of disease (Gautret and Steffen 2016).

At the same time, many of these events are of great cultural importance and suppression of them is neither a realistic nor a desirable option. There have been extensive discussions of how to address the public health needs of the great numbers of people who undertake pilgrimages or who attend other events that draw great numbers of people together. Vaccination may create herd immunities that reduce risks of disease transmission; for example, for this year’s Hajj the Saudi Arabian government is requiring proof of a quadrivalent Meningococcal vaccination in order to receive a visa (Ministry of Hajj 2017). Nonetheless, risks may remain significant for conditions that cannot currently be addressed by vaccination or that are difficult to treat, such as Middle East respiratory syndrome coronavirus (MERS-CoV) or resistant infections. Information too is critical: such well-attended events require imaginative and thoughtful surveillance that informs short-term medical care. Because Saudi Arabia has had the largest number of human cases of MERS-CoV—an estimated 80% (WHO 2017b)—travelers for this year’s Hajj are being warned to take extra precautions with respect to sanitation and personal hygiene measures such as handwashing or avoiding direct contact with non-human animals (New Zealand Ministry of Foreign Affairs 2017).

Still other social factors may contribute to the development of resistant disease that can be identified through surveillance. Given the difficulties for women in Saudi Arabia to see physicians without being escorted, it is understandable that in Saudi Arabia many community pharmacies will dispense antibiotics without a prescription. Zowasi (2016) recommends addressing these issues by increased education especially through social media as to the best approach to respond to the risk of anti-microbial resistant organism.

Still other recommendations about information use involve research on the development of new forms of antimicrobials. According to the most recent review article (Butler et al. 2017), antibiotics “are dramatically undervalued by society, receiving a fraction of the yearly revenue per patient generated by next-generation anticancer drugs.” They are in the judgment of these authors an “endangered species,”—but there is some faint encouraging news. WHO and a number of national governments have recently begun to direct attention to the potential threat of resistance and lack of new drugs. Since 2000, five new-in-class antibiotics have been marketed, but these unfortunately only target gram-positive organisms not the gram-negative organisms that are likely to be resistant. Other compounds are also in various stages of the process of clinical trials, but these too are more likely to be active against gram-positive bacteria. In the judgment of the authors of this review article, “the acute positive trend of new approvals masks a chronic underlying malaise in antibiotic discovery and development.” Interest in antibiotic development is more likely to be present in smaller biotech companies and in biotech companies located in Europe. The authors conclude: “The only light on the horizon is the continued increase in public and political awareness of the issue.” They also observe that with the retrenchment in investment, “we potentially face a generational knowledge gap” and drug development “is now more important than ever.”

To address this perilous juncture in antimicrobial research, the Pew Charitable Trust convened a scientific expert group in 2016. The premise of the group was that regulatory challenges, scientific barriers, and diminishing economic returns have led drug companies largely to abandon antibiotic research—yet antimicrobial resistance is accelerating. No entirely new classes of antibiotics useful against resistant organisms have been brought to market that are not derivatives of classes developed before 1984—over 30 years ago. The Pew report advances many explanations for this dismal situation, including importantly the lack of coordinated investment in the relevant basic and translational research. One aspect of the report detailed the major role played by information gaps. Published research is out of date and out of print. Moreover, in today’s world of investment in drug discovery, “creating an environment in which data exchange and knowledge sharing are the status quo will be difficult given proprietary concerns and the variety of information types and formats, which may range from historical data to new findings produced as part of this research effort.” The Pew consensus is that the following forms of information sharing are needed: a review of what is known about compounds that effectively penetrate gram-negative bacteria, a searchable catalogue of chemical matter including an ongoing list of promising antibacterial compounds, information on screening assays and conditions tested, and an informational database of available biological and physicochemical data. Mechanisms must also be developed for sharing drug discovery knowledge in the area (Pew, pp. 19–20).

In line with Pew, a European antimicrobial resistance project suggests that research is seriously underfunded (Kelly et al. 2015). This group argues that the bulk of the publicly funded research is in therapeutics (63%); among the remainder, 14% of the research was on transmission and only 3% specifically on surveillance. This group also concluded that research is not coordinated and there is little attention to data sharing or sharing of research results. Funding is fragmented, too, with many smaller grants addressing smaller projects independently rather than in a way that builds. This group summarizes: “to conclude, investment at present might not correspond with the burden of antibacterial resistance and the looming health, social, and economic threat it poses on the treatment of infections and on medicine in general. Antibacterial resistance clearly warrants increased and new investment from a range of sources, but improved coordination and collaboration with more informed resource allocation are needed to make a true impact. Hopefully, this analysis will prompt nations to pay due consideration to the existing research landscape when considering future investments.”

Additional recommendations from other groups include novel methods for management of resistant disease, such as addressing the intestinal microbiome (e.g. Bassetti et al. 2017); these methods, too, may be furthered by surveillance information as well as information about individual patients.

Analysis of these uses of information from the perspective of vector or victim are, we now argue, incomplete.

## The Vector Perspective

When contagious diseases are serious or highly likely to be fatal and treatments for them are limited at best, fear is understandable. Fear may be magnified if the disease is poorly understood, especially until modes of transmission have been identified. Fear may also be magnified if there are no known effective treatments for the disease, as may be the case for extremely drug resistant infections. It is therefore understandable that proposals may come to the fore that emphasize isolation of those who are known to be infected, quarantine of those who have been exposed, or travel bans from areas of known disease outbreaks. Proposals may even include criminalization of those who knowingly or even negligently take risks of infecting others.

All of these possibilities and more were features of the HIV epidemic. Even as understanding of the disease grew and effective treatment became increasingly available, some of these remain. Criminalization of HIV transmission has not waned, despite the many objections raised to it (e.g. Francis and Francis 2013a, b). Although the US ended its immigration ban on HIV+ individuals in 2010, concerns remain about the risks of undiagnosed infections among immigrant populations in the U.S. (Winston and Beckwith 2011) and some countries (for example, Singapore) continue to ban entry for HIV+ travelers planning stays over thirty days (The Global Database 2017).

As epidemic fears have waxed and waned over recent decades, so have imperatives for identifying vectors and constraining their activities. These patterns have been apparent for avian influenza, SARS, Ebola, and Zika, among others. The US still bars entry by non-citizens with a list of conditions including active TB, infectious syphilis, gonorrhea, infectious leprosy, and other conditions designated by Presidential Executive order such as plague or hemorrhagic fevers (CDC 2017).

Indeed, resistant TB has been a frequent illustration of the vector perspective in operation. Multi-drug resistant tuberculosis is transmissible, difficult to treat, and poses a significant public health problem. Its presence can be identified by methods such as testing of sputum samples. When patients are identified with resistant disease, public health authorities may seek to compel treatment or isolation, especially for patients judged unreliable about compliance with treatment. To avoid transmission, public health authorities have proposed isolating patients who have been identified as infected. Because a course of treatment for TB may take many months—and failure to complete the full course may increase the likelihood of resistant disease—isolation may continue for long periods of time. Controversially, during the early 1990s public health officials in New York isolated over 200 patients identified with MDR TB on Roosevelt Island for treatment out of concern that they would be non-compliant with treatment even when they were unlikely to infect others (Coker 2001).

Perhaps one of the most highly publicized events involving a single patient was the odyssey of Andrew Speaker, a lawyer believed to have extremely resistant TB who eluded authorities as he took airplane flights around the globe in the effort to return home. Speaker’s journey created an international scare and calls for travel restrictions. Speaker’s lawsuit against the Centers for Disease Control and Prevention alleging violations of the federal Privacy Act, he claimed by revealing more information than was necessary for public health purposes, was ultimately resolved on summary judgment for the government, largely because the challenged disclosures had been made by Speaker himself. (*Speaker v. U.S. Department of Health and Human Services Centers for Disease Control and Prevention*, 489 Fed. Appx. 425 (2012); Associated Press 2010) But the saga reveals how individuals perceived as threats may be vilified for what were understandable, if unwise, efforts at self-protection.

WHO travel guidelines provide that individuals known to be infected with resistant TB should not travel until sputum analysis confirms that they are not at risk of disease transmission (WHO 2017). Evidence is limited, however, about the need for this policy. The most recent literature review suggests that risks of transmission during air travel are very low and that there is need for ongoing international collaboration in contact tracing and risk assessment (Kotila et al. 2016). Blanket travel bans encouraging actions that elude detection may reduce, rather than enhance, this needed collaboration. More subtle policies tailored to need would be preferable, but the fears generated by a focus on fear of vectors may make them unlikely to be developed or implemented.

At best, therefore, the vector perspective is incomplete. Focus on it may be counter-productive, if people hide or try to avoid education. It may encourage expenditures on efforts to identify suspected vectors rather than on evidence based efforts to identify risks of transmission and effective modes of prevention. And, of course, it ignores the plight of victims, to which we now turn.

## The Victim Perspective

People with resistant infections are not only vectors, they are also victims of disease and have ethical claims to be treated as such (Battin et al. 2007). Indeed, it is likely that vectors will themselves be victims, unless they are carriers of the disease in a manner that does not affect them symptomatically.

Concern for victims may take the form of seeking to ease the burdens of constraints such as isolation. A good illustration of the victim perspective in operation is the WHO publication of a pamphlet on “psychological first aid” to those affected by Ebola. The pamphlet is designed to provide comfort to and meet the basic needs of people infected by Ebola and those who are close to them, while maintained the safety of aids workers (WHO 2014). The recommendations rest on the importance of respect for the dignity of those who are suffering amidst disease outbreaks. It also emphasizes the importance of respect for rights such as confidentiality and non-discrimination. The pamphlet is provisional and designed to be updated as knowledge of safety measures improves; this provisional nature is a recognition of the importance of ongoing development of information about how victims’ needs can be safely met.

Despite the concern for victims, foremost in the pamphlet’s recommendations is safety, both of aid workers and of disease victims, so that no one is further harmed including victims themselves and others close to them. Overall, the pamphlet attempts to counter impulses to come to the aid of victims that may increase transmission risks, such as unprotected contact with those who are ill. But unexplored tensions remain in the document’s recommendations. For example: “Respect privacy and keep personal details of the person’s story confidential, if this is appropriate” (p. 22). Nowhere does the document discuss when confidentiality is appropriate or what personal details may be revealed and in what ways. Its manifest and important concern for victims is countered by safety but without discussion of how these goals might be implemented together or reasonably reconciled in practice.

The WHO’s most recently-adopted strategy for dealing with health emergencies, the Health Emergencies Programme, provides another illustration of concern for victims that may lie in unexplored tension with other values. The Programme urges cooperative methods to meet the immediate health needs of threatened populations through humanitarian assistance while also addressing causes of vulnerability and recovery (WHO 2016). It is a coordinated strategy for emergency response that will move far beyond merely technical help; WHO describes it as a “profound change for WHO, adding operational capabilities to our traditional technical and normative roles” (WHO 2016). It is aimed to provide crisis help, such as to Hurricane Matthew in Haiti or to areas affected by the Zika virus. It requires a major increase in funding devoted to core emergency efforts. Core funding will come from assessed contributions, flexible contributions that the Director-General has discretion to allocate, and earmarked voluntary contributions. But it is clearly under-funded; WHO reported a 44% funding gap as of October 2016, just to meet the program’s core capabilities. Moreover, WHO also reported that it has raised less than a third of the funding needed for the WHO Contingency Fund for Emergencies, a fund deployed for the initial 3 months of an emergency before donor funding becomes available (WHO 2016).

The Health Emergencies Programme reflects reactions to the humanitarian disaster of the Ebola epidemic and criticisms of the WHO level of response. The WHO 2016–2017 budget reflects this response as well (WHO 2015). That budget “demonstrates three strategic shifts” (WHO 2015, p. 2). The first is application of the lessons from Ebola especially the need to strengthen core capacities in preparedness, surveillance and response. The second strategic shift is a focus on universal health coverage, which includes enhancing contributions to maternal and child health, speeding progress towards elimination of malaria, and enhancing work on non-communicable diseases, among other worthy goals. The final strategic shift is towards “emerging threats and priorities”; illustrations of these are “antimicrobial resistance, hepatitis, ageing, and dementia.” These are not an obvious group to characterize as “emerging,” to the extent that this suggests a developing threat that has not yet become urgent but that may be expected to become so in the near future. Nor are they an obvious group to link together in the same category. This mixture of budgetary priorities suggests is responsiveness to issues raised through consultation with WHO member states, rather than proactive planning.

WHO specific efforts directed to resistance can be characterized as primarily coordination. The WHO website devoted to resistance promotes information sharing and lists research questions and potential funding agencies (WHO 2017a). WHO expresses no judgment about either funding agencies or which of the nearly 100 listed research questions—ranging from research on resistance in day care centers to the biological price that microorganisms pay for resistance—might be fruitfully addressed first or how they might be interconnected.

Concern for victims is surely part of a response to a humanitarian emergency. Responsiveness to urgent health needs is an important goal. Including antimicrobial resistance in a list of “emerging” issues is at least recognition of the problem. But the WHO response to Ebola and the WHO budget overall can be characterized as less than fully set into context in a reasoned way.

Thus, we contend, neither vector nor victim perspectives are adequate. One risks falling prey to fear while the other risks responses that are well-intentioned but that may be difficult to meet or compete with other values in ways that remain underexplored. These perspectives are inevitable and important, but they are each incomplete.

##  Fairness in Information Use

In our judgment, a primary difficulty with both vector and victim perspectives is that neither are set into context or seen as interconnected. This section suggests how fairness considerations may help in focusing attention to the most pressing questions to ask about antimicrobial resistance and the directions for surveillance and information use to take.

 Fairness entered the philosophical lexicon in discussions about justice as procedural, most famously in John Rawls’s “ Justice as Fairness” (Rawls 1958). As Rawls initially conceptualized his view, it involved a decision procedure for selecting basic principles of justice in which people were unable to gain unfair advantage. As the debates about Rawlsian justice unfolded, a fundamental issue was whether people with radically different capacities and views of the good life could be expected to accept the results of the decision procedure as formulated. Thus critics raised the concern that people with disabilities might be left out of the decision procedure as “non-contributors” to the practice of justice (Nussbaum 2007; Stark 2007). Critics also pressed the argument that people with radically illiberal conceptions of the good would ultimately destabilize the practice of justice in a Rawlsian ideal society (e.g. Williams 2007a, b). Rawls ultimately accepted the point that proceduralism could not yield a universal theory of justice, pulling back his view to the claim that it only represented a vision of justice for a certain kind of liberal society (Rawls 1993).

But fairness also entered the debates about justice in a more substantive way, especially in bioethics. Norman Daniels (1985), for example, expanded a Rawlsian approach to consider justice in health care. The British idea of a “fair innings,” in which the opportunities of each to reasonable health over a normal life span are prioritized, was raised particularly with respect to the distribution of health care resources to the elderly (Bognar 2015; Farrant 2009; Harris 1985; Williams 2007b). Like the metaphor of a level playing field, the fair innings argument comes from sports (Francis 2017). It reflects the idea of everyone having a chance to participate in a game that at least gives them a reasonable opportunity for success. There are four aspects of such opportunity: who plays and whether the rules are constructed to give each an opportunity to win that is reasonable are two. Also important is the balance among opportunities to succeed, so that there aren’t consistent tilts in one direction or another, as might be characterized by the further metaphor of leveling the playing field. Finally, attention to the interaction between advantages and disadvantages matters, so that participants are encouraged to continue playing the game rather than dropping out.

Our invocation of fairness as a concept is rooted in the judgment that antimicrobial resistance—or other pressing global public health problems, for that matter—exemplify multiple aspects of non-ideal and partial compliance circumstances. Natural circumstances are less than forgiving; new health threats emerge on a regular basis. Antimicrobial resistance is an ongoing natural challenge to effective therapy for deadly diseases. Social circumstances are imperfect, too: overcrowding, poor sanitation, straitened resources for public health and health care, and cultural practices that increase potential for disease transmission all play roles in the development of resistance. Alexander Fleming, the discoverer of penicillin, warned that the development of resistance was likely, but his warning appears not to have been well heard. Finally, efforts to address antimicrobial resistance are riven with non-compliance: over-prescribing by physicians, over-use of antimicrobials in agriculture, individual failures to take medications as prescribed, and concealment of disease out of fear of discovery and persecution. Because the conditions that give rise to these problems of non-compliance may seem urgent—people seeking antimicrobials are in pain or ill, perhaps gravely; people in hiding from health authorities may fear stigmatization or death—they raise in particularly poignant form questions of the extent of obligations under circumstances in which others are not doing what arguably is their fair share (e.g. Stemplowska 2016; Murphy 2000).

 Fairness as an ethical concept is especially suited to such imperfect circumstances. It directs attention to how improvements are distributed. Distributions can be more or less fair, if they distribute benefits and burdens in an increasingly inclusive manner (e.g. Francis and Francis 2013a, b). Fairness thus construed is at the heart of perhaps the most influential set of recommendations for ethical pandemic planning, the Canadian *Stand on Guard for Thee* (Toronto Joint Centre 2005). Although much of the discussion of fairness in this document emphasizes inclusive procedures, so that engagement may lead to acceptance of choices as fairly made (e.g., p. 1), the recommendations also contain substantive dimensions. These include fair resource plans for those who fall ill providing necessary services during a pandemic (p. 11) and assurance that people who are affected by choices are reciprocally supported in a way that they do not suffer “unfair economic penalties” (p. 13). Here, the links between fairness and reciprocity are explicit.

These four aspects of fairness—who is included in the play, what opportunities they have, how these opportunities are balanced, and whether there are elements of reciprocity—can be used to set vector and victim perspectives into context in addressing the gathering and use of information about antimicrobial resistance. Over-emphasizing vectors threatens their opportunities and even possible participation. Overemphasizing victims tilts the field unidirectionally, understandably directing resources to immediate need but without consideration of longer-term consequences. Reciprocity may be the most important of all, creating commitment to workable strategies for addressing resistance when there are difficult choices to be made.

Fear, understood as a threat personal health, is often an ally in persuading people to seek preventive care and to change life styles, or to persuade policy makers to create incentives or penalties for decisions that contribute to poor health. But great fear can also lead to immobility. The real threat posed by the rise of antimicrobial resistance does not seem to be easily addressed by a successful alternative in the view of victims or policy makers. Medical personnel are fearful of not responding to the demands of patients for immediate reductions in pain or suffering at relatively low costs. The scale of the threat posed by rapid rise of antimicrobial resistance may be daunting to policy makers especially as funders of research. The cost of developing ever-new generations of antibiotics seems to suggest a great series of short-term solutions especially as pharmaceutical companies respond to incentives to generate near-term profits. In this context, it is worth recalling how the development of the first antimicrobials contributed to more generally shared benefits: when penicillin became known to people as a wonderful drug it actually helped to speed the adoption of the National Health Service in Britain. The popular expectation was health care for all facilitated with the rise of a new generation of low cost wonder drugs and reinforced by low cost vaccinations (Webster 2002). But some of the advantages were short-lived, as the costs of pharmaceuticals grew exponentially and inadequate attention was paid to the risks of overprescribing—once again a cautionary reminder of the importance of emphasizing balance rather than one particular perspective such as victimhood. If a promise of sustaining production at lower costs of ever-new generations of antimicrobials from how information is used can offer benefits more widely, then it becomes easier to impose tougher regulations on anti-microbial use that may to some extent stave off the development of resistance.

This approach in terms of fairness directs attention not only to vectors and to victims seen as separate entities. It also directs attention to how they are often, and unpredictably, twinned—given the epidemiology of resistance spread, it is likely to begin within interlaced communities where vectors are also victims. But it also directs our attention to these issues set in distributive context, raising questions such as these: Who is most likely to be affected by resistance? Who will suffer the most severe consequences from resistance? Who is most likely to be disadvantaged by information gained to counter resistance? Who will suffer the most severe disadvantage? Who will benefit from efforts to counter resistance? How can these benefits be spread more inclusively? And, how are the benefits and burdens of addressing resistance intertwined? Are some primarily beneficiaries, while others are primarily burdened? Are there ways to increase reciprocal linkages in these benefits and burdens, so that efforts to counter resistance are accepted and supported more widely? These are the kinds of questions that need to guide how surveillance is deployed in the effort to counter resistance, not vague generalities about the importance of addressing health infrastructure or bromides about the need to increase resources.
